# Tyrosine-610 in the Receptor Kinase BAK1 Does Not Play a Major Role in Brassinosteroid Signaling or Innate Immunity

**DOI:** 10.3389/fpls.2017.01273

**Published:** 2017-08-02

**Authors:** Vijayata Singh, Artemis Perraki, Sang Y. Kim, Stuti Shrivastava, Jae H. Lee, Youfu Zhao, Benjamin Schwessinger, Man-Ho Oh, Amy Marshall-Colon, Cyril Zipfel, Steven C. Huber

**Affiliations:** ^1^Department of Plant Biology, University of Illinois, Urbana IL, United States; ^2^The Sainsbury Laboratory, Norwich Research Park Norwich, United Kingdom; ^3^United States Department of Agriculture, Agricultural Research Service Urbana, IL, United States; ^4^Department of Crop Sciences, University of Illinois, Urbana IL, United States; ^5^Department of Biological Science, College of Biological Sciences and Biotechnology, Chungnam National University Daejeon, South Korea

**Keywords:** receptor kinase, co-receptor, innate immunity, growth, brassinosteroid, pathogen-associated molecular pattern, tyrosine phosphorylation

## Abstract

The plasma membrane-localized BRI1-ASSOCIATED KINASE1 (BAK1) functions as a co-receptor with several receptor kinases including the brassinosteroid (BR) receptor BRASSINOSTEROID-INSENSITIVE 1 (BRI1), which is involved in growth, and the receptors for bacterial flagellin and EF-Tu, FLAGELLIN-SENSING 2 (FLS2) and EF-TU RECEPTOR (EFR), respectively, which are involved in immunity. BAK1 is a dual specificity protein kinase that can autophosphorylate on serine, threonine and tyrosine residues. It was previously reported that phosphorylation of Tyr-610 in the carboxy-terminal domain of BAK1 is required for its function in BR signaling and immunity. However, the functional role of Tyr-610 *in vivo* has recently come under scrutiny. Therefore, we have generated new BAK1 (Y610F) transgenic plants for functional studies. We first produced transgenic Arabidopsis lines expressing BAK1 (Y610F)-Flag in the homozygous *bak1-4 bkk1-1* double null background. In a complementary approach, we expressed untagged BAK1 and BAK1 (Y610F) in the *bak1-4* null mutant. Neither BAK1 (Y610F) transgenic line had any obvious growth phenotype when compared to wild-type BAK1 expressed in the same background. In addition, the BAK1 (Y610F)-Flag plants responded similarly to plants expressing BAK1-Flag in terms of brassinolide (BL) inhibition of root elongation, and there were only minor changes in gene expression between the two transgenic lines as monitored by microarray analysis and quantitative real-time PCR. In terms of plant immunity, there were no significant differences between plants expressing BAK1 (Y610F)-Flag and BAK1-Flag in the growth of the non-pathogenic *hrpA^-^* mutant of *Pseudomonas syringae* pv. *tomato* DC3000. Furthermore, untagged BAK1 (Y610F) transgenic plants were as responsive as plants expressing BAK1 (in the *bak1-4* background) and wild-type Col-0 plants toward treatment with the EF-Tu- and flagellin-derived peptide epitopes elf18- and flg22, respectively, as measured by reactive oxygen species production, mitogen-activated protein kinase activation, and seedling growth inhibition. These new results do not support any involvement of Tyr-610 phosphorylation in either BR or immune signaling.

## Introduction

Receptor kinases (RKs) play important roles in perceiving extracellular signals and activating downstream signaling via auto- and trans-phosphorylation reactions (Clouse, 2011). Plant RKs constitute a monophyletic group that also includes the receptor-like cytoplasmic kinases (RLCKs) that together are classified as serine/threonine protein kinases. The plant RKs are structurally similar to animal receptor tyrosine kinases but are evolutionarily distinct (Shiu and Bleecker, 2001). Animal receptor tyrosine kinases primarily phosphorylate on tyrosine residues whereas many plant receptor kinases that display kinase activity can phosphorylate on serine and threonine residues (Mitra et al., 2015); however, some plant RKs have dual specificity capable of phosphorylating on serine, threonine and tyrosine residues (Clouse, 2011; Jaillais et al., 2011; Clouse et al., 2012; Lin et al., 2014; Macho and Zipfel, 2014; Macho et al., 2014, 2015; Nemoto et al., 2015; Suzuki et al., 2016). Although now well-recognized, one of the first studies to identify dual specificity was with BRI1, the receptor kinase that initiates brassinosteroid signaling. Using the recombinant cytoplasmic domain of BRI1, autophosphorylation on tyrosine residues was identified and Tyr-831, located in the juxtamembrane domain, was established as a major site of autophosphorylation both *in vitro* and *in vivo* (Oh et al., 2009). Subsequently BAK1, which functions as a co-receptor kinase with BRI1 in BR signaling, and with FLS2 in flg22 signaling (Ma et al., 2016), was also shown to have dual specificity and Tyr-610 in the carboxy-terminal domain was identified as a major phosphotyrosine site (Oh et al., 2010). Sequence- and modification-specific antibodies were developed to monitor phosphorylation at the Tyr-610 site with the recombinant cytoplasmic domain of BAK1 that was autophosphorylated *in situ* during expression in *Escherichia coli*. Furthermore, studies with previously produced and characterized transgenic plants expressing BAK1-Flag in the *bak1-4 bkk1-1* double null mutant background (Wang et al., 2008) established that phosphorylation of Tyr-610 occurs *in vivo* in a BL-dependent manner. A specific role for Tyr-610 *in vivo* was suggested by a dramatic phenotype of transgenic plants expressing the BAK1 (Y610F) directed mutant, which were dwarfed and resembled BR signaling mutants. However, in continuing studies with the BAK1 (Y610F)-Flag transgenic plants, we came to realize that the plants generated in the original study were not correctly genotyped, resulting in retraction^1^ of the original publication.

Because conclusions about the physiological role of BAK1 Tyr-610 had not been properly established, we generated new transgenic plants expressing BAK1 (Y610F)-Flag driven by the native promoter in the double null mutant *bak1-4 bkk1-1* background. The homozygous *bak1-4 bkk1-1* double mutant is seedling lethal (He et al., 2007) but can be rescued by expression of functional BAK1. This system was used previously to determine the functional role of various serine and threonine phosphosites in BAK1 (Wang et al., 2008) and was used in the present study to elucidate the role of Tyr-610 *in vivo*. In addition, the untagged BAK1 (Y610F) transgene driven by the BAK1 native promoter was also transformed in the *bak1-4* mutant background, as it was recently shown that the presence of a carboxy-terminal tag on BAK1 can interfere with its function in immunity (Ntoukakis et al., 2011). Previous studies have shown that the *bak1-4* homozygous mutant exhibits reduced leaf growth, shorter hypocotyls in the dark and partial BL insensitivity in the root-growth inhibition assay (Kemmerling et al., 2007). Experiments with both untagged BAK1 (Y610F) and BAK1 (Y610F)-Flag strongly suggest that Tyr-610 does not have an important role in plant development or innate immunity.

## Materials and Methods

### Plant Growth

Seeds were surface sterilized using 70% ethanol for 1 min and 50% sodium hypochlorite for 8 min, followed by washing with sterilized water. After 2 days of stratification at 4°C, the seeds were plated on half-strength Murashige and Skoog basal salt medium containing 1% sucrose (pH 5.7) and 0.8% agar in a controlled growth chamber with 130 μmol photons (PAR) m^-2^ s^-1^ and a 16 h light/8 h dark cycle at 22°C. After 7 days, seedlings were transferred to moistened soil and grown under the same conditions. The *bak1-4*/BAK1 (Y610F) mutant lines were grown on soil as one plant per pot in controlled rooms maintained at 20°C–22°C with a 10 h photoperiod and assays on those plants were performed about 4 weeks postgermination. The *bak1-4*/BAK1 (Y610F) seedlings used to perform seedling growth inhibition and MAPK activation assays were grown on plates with Murashige and Skoog (MS) media supplemented with vitamins and 1% sucrose (Duchefa) in rooms with a 16 h photoperiod.

### Plasmid Construction and Generation of Transgenic Plants

All mutants and transgenic lines used in this study were in the background of *Arabidopsis thaliana* ecotype Columbia (Col-0). *BAK1* was amplified by PCR from the wild-type cDNA using Pfu DNA polymerase (Agilent Technologies, Santa Clara, CA, United States) and the BAK1 cDNA was cloned into the binary vector pBIB with its native promoter and Flag epitope tag at the C-terminus. The BAK1 Y610F directed mutant was generated using the Stratagene Site-Directed Mutagenesis Kit (Stratagene, La Jolla, CA, United States). The whole cloned product was confirmed by sequencing. Binary vector constructs containing BAK1::BAK1-Flag or BAK1::BAK1 (Y610F)-Flag were introduced into *Agrobacterium tumefaciens* GV3101, which was then used to transform the ‘D21’ heterozygote (*BAK1 bak1-4/bkk1-1 bkk1-1*) using the floral dipping method (Clough and Bent, 1998). The list of primers is given in Supplementary Table 3. Transgenic seedlings (T1) were selected on half-strength MS media (Phyto Technology Laboratories, Lenexa, KS, United States) containing 30 mg L^-1^ hygromycin B (Sigma–Aldrich, St. Louis, MO, United States). Selected T2 seeds derived from individual T1 plants were screened on the same medium to genotype further. Three independent lines of T3 seeds were ultimately obtained and used for all subsequent experiments.

BAK1 and BAK1 (Y610F) were PCR amplified using primers given in Supplementary Table 3 and inserted following BsrgI and BamHI (NEB) digestion and ligation into epiGreenB2 vector containing the whole genomic region of BAK1, including a 1445 bp promoter fragment promoter fragment (Schwessinger et al., 2011). Resulting constructs pBAK1::cBAK1 (Y610F) were transformed into *A. tumefaciens* strain AglI containing the pSOUP helper plasmid, which was then used for transformation of the *bak1-4* (He et al., 2007) plants by floral dipping. Homozygous lines carrying a single transgene insertion were selected, and T3 homozygous plants were used for detailed phenotypic characterization.

### DNA Extraction and Genotyping of Transgenic Plants

Genomic DNA (gDNA) was isolated from Arabidopsis leaves for use as template in PCR. Fresh leaves were homogenized in DNA extraction buffer containing 200 mM (Tris-HCl, pH 8.0, 250 mM) NaCl, 0.5% SDS, and 25 mM EDTA. Samples were incubated for 10 min at room temperature. About 150 μL of phenol: chloroform (1:1) was added to the extract followed by incubation for 5 min at room temperature. The mixture was then centrifuged at ∼6,000 *g* for 10 min. The upper aqueous layer was removed and an equal volume of isopropanol was added to precipitate gDNA. The precipitated gDNA was washed with two volumes of 70% ethanol and the dried pellet was dissolved in sterile water. About 80 ng of gDNA has taken for each PCR reaction. Alternatively, the genotyping of the *bak1-4*/BAK1 (Y610F) lines was performed using the Whatman FTA card technology (GE Healthcare). The list of primers used is given in Supplementary Table 3.

### Isolation of Microsomal Membrane and Immunoprecipitation of BAK1-Flag and Immunoblot Analysis

Sterilized seeds were grown in liquid culture with continuous shaking (∼70 rpm) under long day conditions (16 h light/8 h dark) for 10 days. Seedlings were collected, blotted dry, and ground to a powder in liquid nitrogen. Frozen powdered tissue was ground in a mortar with twice the volume of protein extraction buffer containing 50 mM Tris-HCl, pH 7.5, 100 mM NaCl, 250 mM mannitol, 5 mM EDTA, 10% glycerol, 0.5% (w/V) polyvinylpolypyrollidone, protease inhibitors (Research Products International, Mount Prospect, IL, United States) along with 1 g of acid-washed sand. The resulting extract was filtered through Miracloth (Calbiochem, La Jolla, CA, United States) and then centrifuged at 15,000 *g* for 30 min to pellet debris. The supernatant was removed and the microsomal fraction was then isolated by centrifugation at 100,000 *g* for 60 min at 4°C. The microsomal pellet was resuspended in protein extraction buffer containing 1% (v/v) Triton X-100 (hereafter referred to as binding buffer) and BAK1-Flag was immunopurified using anti-Flag M2 affinity beads (Sigma–Aldrich, St. Louis, MO, United States) with overnight gentle shaking in the cold room. The beads were washed three times with binding buffer containing 50 mM Tris-HCl, pH 7.5 and 250 mM NaCl. Protein was eluted from the beads with a double volume of 2X SDS-PAGE sample buffer, followed by SDS-PAGE, electrophoretic transfer to PVDF, and immunoblotting with anti-Flag antibodies. Immunoblots were scanned using an Odyssey Infrared Imaging System (LI-COR Bioscience, Lincoln, NE, United States).

### Gene Expression by Microarray Analysis

Plants were grown under long day conditions (16 h light/8 h dark) for 12 days on half-strength MS agar plates. Seedlings were harvested in liquid nitrogen, and stored at -80°C. Total RNA was isolated and cleaned using the RNeasy Plant Mini Kit (Qiagen, Valencia, CA, United States). Total RNA was extracted in triplicate from pooled samples of several plants expressing BAK1-Flag or BAK1 (Y610F)-Flag, and Agilent Gene Expression microarray analysis was performed by the W. M. Keck Center for Comparative and Functional Genomics at the Carver Biotechnology Center, University of Illinois. RNA was labeled and hybridized to the Agilent Arabidopsis V4 array using the Agilent Low-Amp Gene Expression Two Color Labeling kit according to the manufacturer’s protocols. In brief, 200 ng of total RNA was denatured at 65°C for 10 min followed by 5 min on ice. Reverse transcription was carried out at 4°C for 2 h followed by a 15 min incubation at 70°C to inactivate enzymes. *In vitro* transcription was then carried out while incorporating Cy3- or Cy5-labeled nucleotides to create labeled RNA. Labeled RNA was purified using the Qiagen RNA easy kit and the purified product was fragmented and hybridized at 65°C for 17 h. Following hybridization, slides were washed in Agilent wash buffers and scanned on an Axon 4000B microarray scanner. Spot finding was carried out using GenPix 6.1 image analysis software. Differentially expressed genes (DEGs) were determined using Rank Product analysis (Breitling et al., 2004) (RP function in R Bioconductor) and considered significantly different at *p*-value ≤ 0.1 (FDR-correction at 5%). Subsequent [log_2_] fold change analysis was performed. Gene Ontology (GO) analysis was performed using the Biomaps function in VirtualPlant (Katari et al., 2010).

### Gene Expression Analysis by Quantitative Real-Time PCR (qPCR)

Total RNA was extracted from 12-day-old Arabidopsis seedlings (grown as described above for microarrays) with the EasyPure Plant RNA Kit (TransGen Biotech, Beijing, China). One microgram of total RNA was used to synthesize cDNA with the oligo-(dT) 18 primer using the Verso cDNA synthesis KitSuperMix (Thermo Fisher Scientific). Quantitative real-time PCR analysis of cDNA was performed on a Light Cycler 96 Roche using Real Master Mix (SYBR Green) (Fast Start Essential DNA Green Master Mix) and the specific primers. The following thermal cycle condition was used: 95°C for 2 min, followed by 45 cycles of 95°C for 20 s and 55°C for 20 s, 72°C for 30 s. All reactions were performed in triplicate on three biological replicates (three plants per sample). Relative quantification of specific mRNA levels was analyzed using the cycle threshold (Ct) 2^-ΔΔCt^ method, normalized using the housekeeping gene Actin-2. The list of primers used are listed Supplementary Table 3.

### Hypocotyl Elongation Assay

To examine the effect of BL on the growth of hypocotyls, seeds were surface-sterilized, cold-stratified for 2 days, and then placed on MS media agar plates under long day conditions for 24 h to induce germination. After the medium was exchanged for a medium supplemented with various concentrations of BL, seedlings were further grown vertically in darkness for 7 days. After 7 days plates were taken out and hypocotyls were measured.

### Bacterial Infection Assays

*Pseudomonas syringae* pv. tomato (*Pst*) DC3000 *hrpA^-^* was grown overnight in LB medium containing rifampicin (25 mg L^-1^) and kanamycin (50 mg L^-1^) at 28°C on a shaker (250 rpm). Plants were inoculated by infiltrating three lower leaves of 5-week-old plants with a suspension of *Pst* DC3000 *hrpA^-^* (10^4^ cfu mL^-1^) in PBS using a needle-less syringe. For mock inoculation, PBS was used for infiltration. On the third day after infiltration, leaves were harvested and 5 mm leaf disks from five infiltrated leaves were taken using a cork borer; the leaf disks were ground in 1 mL of PBS and 10^-2^, 10^-3^, and 10^-4^ dilutions were prepared. Plating was done with 25 μL of the dilutions in LB plates containing appropriate antibiotics. Plates were incubated in 28°C chambers and after 2 days, bacterial colonies were counted manually from the plate with an appropriate dilution. From the manual counts, CFU per leaf disk was calculated using the equation: CFU/Leaf disk = [1000/(4 × 25)] (number of colonies) (dilution).

### Oxidative Burst Assay

Twelve leaf disks (4 mm diameter) per genotype were collected in 96-well plates and allowed to recover overnight in 100 μL sterile water. The next day, the water was replaced with an eliciting solution containing 17 mg/mL luminol (Sigma–Aldrich), 10 μg/mL horseradish peroxidase (Sigma–Aldrich), and 100 nM flg22 (Felix et al., 1999) or elf18 (Kunze et al., 2004). Luminescence was recorded over a 60-min time period using a Photek camera (Ltd, East Sussex, United Kingdom).

### MAPK Activation Assay

Seeds were surface sterilized, sown on 1% MS agar plates and stratified for 2 days at 4°C in the dark, before moving them to light. Five days later, seedlings were transferred as two seedlings per well in 24-well plates containing liquid MS media and left for 10 more days. The MS was then removed and the seedlings were elicited by adding fresh MS alone (mock) or containing 100 nM flg22 or elf18 for 5 min, followed by fast freeze in liquid nitrogen. Protein extraction buffer (50 mM Tris, pH 7.5, 1 mM EDTA, pH 8, 150 mM NaCl, 10% [v/v] glycerol, 1% [v/v] IGEPAL CA630, 10 mM dithiothreitol, 1x protease inhibitor cocktail (Sigma–Aldrich), 100 nM calyculin A (New England Biolabs), 2.5 mM Na_3_VO_4_ and 1 mM phenylmethylsulfonyl fluoride) was added to liquid nitrogen-ground samples in a ratio of 2 mL extraction buffer/g of tissue and left for 20 min at 4°C for solubilisation. The soluble extracts were cleared by centrifugation (10 min, 13,000 rpm, 4°C) and then normalized to 10–15 μg of total proteins using Bradford Reagent (Bio-Rad) for western blot analysis, using the anti p44/42-ERK antibody (1:2000) and anti-BAK1 antibodies (1:4000) as previously described (Schwessinger et al., 2011).

### Seedling Growth Inhibition Assay

Seeds were surface sterilized, sown on 1% MS agar plates and stratified for 2 days at 4°C in the dark, before moving them to light to germinate. Five-day-old seedlings were then transferred individually to single wells in 48-well plates containing liquid MS with and without 10 and 100 nM flg22 or elf18. Ten days later, dry weight of eight replicates per treatment was measured using a precision scale (Sartorius) and plotted relative to untreated control.

## Results and Discussion

**FIGURE 1:**
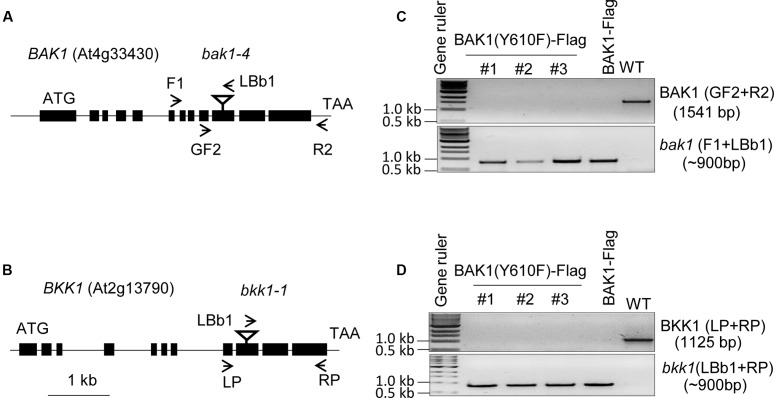
Genotyping of BAK1 (Y610F)-Flag lines in *bak1-4 bak1-4/bkk1-1 bkk1-1* background. **(A,B)** Genomic organization of the T-DNA insertion knockout lines, *bak1-4* (SALK_116202) and *bkk1-1* (SALK_057955), both in the Col-0 ecotype. The position of each T-DNA insertion is depicted by an inverted triangle, and arrows showing positions of primers used for genotyping. **(C)** PCR using *BAK1* gene specific primers (upper gel panel) and a T-DNA specific primer (lower gel panel). **(D)** PCR using *BKK1* gene specific primers (upper gel panel) and a T-DNA specific primer (lower gel panel). The three lines used for genotyping are independent lines; WT is wild-type Col-0 that served as a positive control for gene specific primers and negative control for T-DNA specific primers.

To analyze the role of BAK1 Tyr-610 *in vivo* we expressed the site-directed mutant BAK1 (Y610F)-Flag driven by the native promoter in the double null *bak1-4 bak1-4/bkk1-1 bkk1-1* mutant background. Because seedlings with the double null mutation are not viable (He et al., 2007), we transformed the heterozygous *BAK1 bak1-4*/*bkk1-1 bkk1-1* (D21) line and then selected for presence of the transgene and absence of the endogenous genes. Positive transformants were selected on hygromycin plates and genotyping was done with T1 lines. In the T2 generation, three independent transgenic lines were selected and genotyped and results are presented in **Figure 1**. The *bak1-4* and the *bkk1-1* mutants contain T-DNA insertions in exon 10 and exon 9 of the genes, respectively (**Figures 1A,B**). To identify the presence of the endogenous *BAK1* gene we used *BAK1* gene specific primers (GF2 from exon 9 and R2 from the 3′ UTR) flanking the T-DNA insertion site, and to identify the T-DNA insertion within the *BAK1* gene we used a T-DNA specific primer (LBb1 T-DNA left border) and an upstream primer from *BAK1* (F1 from intron 6; **Figure 1A**). Similarly, to identify the endogenous *BKK1* gene we used gene specific primers (LP from exon 8 and RP from exon 11), and to identify the T-DNA insertion within the *BKK1* gene we used a T-DNA primer (LBb1 T-DNA left border) and RP from exon 11 of *BKK1* (**Figure 1B**). The three independent BAK1 (Y610F)-Flag transgenic lines selected were positive for the *bak1-4* (**Figure 1C**) and the *bkk1-1* T-DNA insertion (**Figure 1D**), and thus are devoid of *BAK1* and *BKK1* transcript (He et al., 2007; Kemmerling et al., 2007). The three transgenic lines expressing BAK1 (Y610F)-Flag lack the 3′-UTR associated with the endogenous gene and thus the transgene was not amplified using the primers employed in **Figure 1C**.

**FIGURE 2:**
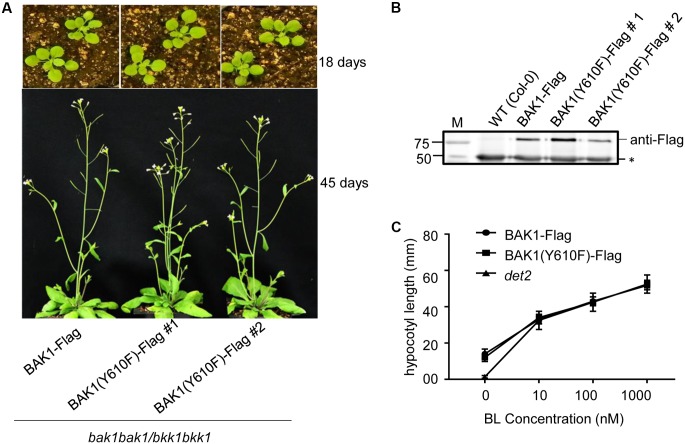
Similar growth and BL responses in transgenic plants expressing BAK1-Flag or BAK1 (Y610F)-Flag in the double null background. **(A)** Phenotype of 18- and 45-day-old Col-0, BAK1-Flag and BAK1 (Y610F)-Flag plants (in the *bak1-4 bak1-4/bkk1-1 bkk1-1* double null background) grown in a normal long day photoperiod. **(B)** Immunoblot analysis indicates similar expression levels of the BAK1-Flag transgene in the different genotypes. Seedlings were grown in liquid MS medium for 10 days. Protein was immunoprecipitated using immobilized anti-Flag antibodies of solubilized microsomal membranes, and immunoblotting was performed with anti-Flag antibodies. The reactive band at 50 kDa (asterisk) is the heavy chain of the immobilized M2 antibody released from the Anti-Flag M2 Beads by elution with SDS-PAGE sample buffer containing reductant. **(C)** Dose-dependent hypocotyl elongation of 7 days old seedlings of BAK1-Flag, BAK1 (Y610F)-Flag and *det2* at 0, 10, 100, and 1000 nM BL. Seedlings were grown in the long day photoperiod.

Transgenic lines harboring BAK1 (Y610F)-Flag not only rescued the seedling-lethal phenotype of the homozygous double mutant, but also the growth and development at vegetative and reproductive stages of all the transgenic plants was very similar to that of wild-type plants. Representative photographs of wild-type and Y610F plants at two stages of development when grown in long day conditions are shown in **Figure 2A**. Expression of the transgene in the two independent lines was similar based on immunodetection of BAK1 (Y610F)-Flag in microsomal membranes isolated from the plants (**Figure 2B**). Similar growth between the BAK1 (Y610F)-Flag plants and wild-type plants suggests that BR signaling is not impaired by inability of BAK1 to autophosphorylate at the Tyr-610 site. As a further test of BR signaling, we examined hypocotyl elongation in response to exogenous brassinolide (BL). As a positive control we used the *det2* mutant that is impaired in BR biosynthesis and thus is completely dependent on exogenous BL for growth (Fujioka et al., 1997), which was confirmed as expected (**Figure 2C**). Hypocotyl elongation was increased equivalently in transgenic plants expressing BAK1-Flag and BAK1 (Y610F)-Flag, providing another line of evidence that BR signaling was similar among all the genotypes tested. Collectively, the results suggest that Tyr-610 autophosphorylation is not essential for BAK1-mediated BR signaling in normal growth and development of Arabidopsis.

It was previously reported (Oh et al., 2010) that gene expression was markedly altered in BAK1 (Y610F)-Flag plants, including many genes associated with stress and/or defense responses, which was interpreted to mean that Tyr-610 was not only required for normal BR signaling but also basal defense gene expression. Therefore, we did transcriptome analysis of the properly genotyped BAK1 (Y610F)-Flag and BAK1-Flag plants (both in the *bak1-4 bak1-4/bkk1 bkk1* background) using Agilent Arabidopsis V4 arrays. The RNA for transcript profiling was isolated from 12-day-old seedlings of BAK1-Flag and BAK1 (Y610F) grown aseptically. On the basis of rank product analysis (Breitling et al., 2004) with a false discovery rate of 0.05, 95 genes were differentially expressed including 51 genes that were up-regulated (Supplementary Table 1) and 44 genes that were down-regulated in BAK1 (Y610F)-Flag more than 50% compared to wild-type BAK1-Flag plants (Supplementary Table 2). This is a relatively small number of DEGs and of those the fold changes were relatively small (**Table 1**).

**Table 1:** Differentially expressed genes (DEGs) with largest fold changes in BAK1 (Y610F)-Flag relative to BAK1-Flag plants by microarray analysis using Agilent Arabidopsis V4 Arrays.

Gene locus ID	Gene description	BAK1/Y610F	
		log_2_ FC	FC
**Up-regulated genes in BAK1 (Y610F)-Flag**			
AT4G28520	Cruciferin 3 (CRC, CRU3)	-2.07	0.24
AT5G44120	Cruciferin A (CRA1, CRU1)	-1.90	0.27
AT1G15520	ATP-binding cassette G40 (ABCG40, PDR12)	-1.82	0.28
AT5G35660	Glycine-rich protein family	-1.65	0.32
AT4G12490	Bifunctional inhibitor/lipid-transfer protein/seed storage 2S albumin	-1.22	0.43
AT4G26150	Cytokinin-responsive gata factor 1 (CGA1, GATA22)	-1.15	0.45
AT1G03106	Hypothetical protein	-1.09	0.47
AT5G50600	Hydroxysteroid dehydrogenase 1 (ATHSD1, HSD1)	-1.08	0.47
AT4G26220	*S*-adenosyl -L-methionine-dependent methyltransferases superfamily	-1.06	0.48
AT2G05580	Glycine-rich protein family	-1.04	0.49
**Down-regulated genes in BAK1 (Y610F)-Flag**			
AT1G75830^∗^	Low-molecular-weight cysteine-rich 67 (LCR67)	1.58	3.00
AT3G22231^∗^	Pathogen and circadian controlled 1 (PCC1)	1.03	2.04
AT1G11610	Cytochrome P450, family 71, subfamily A, polypeptide 18 (CYP71A18)	0.91	1.89
AT4G37610^∗^	BTB and TAZ domain protein 5 (BT5)	0.90	1.87
AT3G30720	qua-quine starch (QQS)	0.89	1.85
AT5G39180^∗^	RmlC-like cupins superfamily protein	0.88	1.84
AT4G33070	Thiamine pyrophosphate dependent pyruvate decarboxylase protein	0.87	1.83
AT3G29970	B12D protein	0.86	1.81
AT3G59940	Galactose oxidase/kelch repeat superfamily protein (ATKFB50, KMD4)	0.84	1.80
AT2G44130	Galactose oxidase/kelch repeat superfamily protein (KFB39, KMD3)	0.79	1.73

**FIGURE 3:**
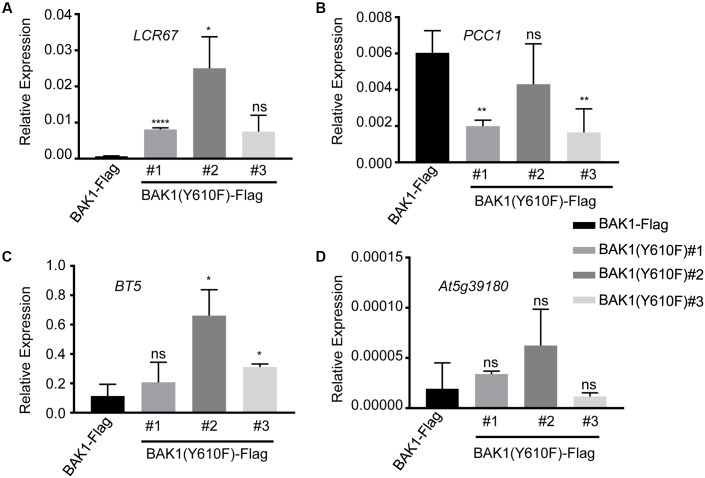
Quantitative real time PCR analysis of genes potentially down regulated in BAK1 (Y610F)-Flag plants relative to BAK1-Flag plants. Transcript accumulation of **(A)** *LCR67*, **(B)** *PCC1*, **(C)** *BT5*, and **(D)** *AT5g39180* in BAK1-Flag and three independent line of BAK1 (Y610F)-Flag. Quantitative real-time polymerase chain reaction was used to determine the abundance of the different transcripts relative to the abundance of the *ACTIN2* transcript. Each data point represents mean ± standard deviation (SD) of *n* = 4. Statistical significance of differences with respect to BAK1-Flag were assessed by Welch test with probability values of ^∗∗∗∗^*p* < 0.0001, ^∗∗^*p* < 0.001, ^∗^*p* < 0.01 and ns, not significant indicated above the respective bars. Experiments were done two times with similar results.

*The asterisk identifies genes selected for qPCR analysis in **Figure 3***.

Several of the genes down-regulated in BAK1 (Y610F)-Flag plants are involved in defense responses and resistance to pathogens, suggesting that there may be some impact on innate immunity. Consequently, we selected four down-regulated genes (identified in **Table 1** with an asterisk following the gene locus ID number) for validation and assessment of absolute expression levels using qPCR analysis. LCR67 (At1g75830) is a defensin-like protein that is a secreted cysteine-rich antifungal protein (Lacerda et al., 2014). PCC1 (At3g22231) is also a cysteine-rich protein containing a transmembrane domain that modulates resistance against pathogens, including fungi, and also affects a number of stress responses including response to UV-C (Sauerbrunn and Schlaich, 2004). BTB/POZ (At4g37610) may act as a substrate-specific adapter for an E3 ubiquitin-protein ligase complex (Geyer et al., 2003) and therefore may mediate the ubiquitination and subsequent proteasomal degradation of selected proteins, and conceivably could play a role in defense responses. Finally, the germin-like protein (Membré et al., 2000), At5g39180, was selected for further study as a secreted Mn-binding protein that likely plays a role in plant defenses. While all four of the selected genes were down regulated in the microarray experiments, only *PCC1* showed the same qualitative difference in expression as the other three genes were up regulated relative to BAK1-Flag when expression was analyzed by qPCR (**Figure 3**). One critical point to note is that all four of these genes had absolute expression levels that were much lower than that of actin, which may explain the lack of correspondence between the microarray results and real-time qPCR analysis. For example, a large-scale comparison of gene expression levels by microarrays and RNAseq documented that low abundance genes generally showed lower correlations between the two expression analysis methods than did genes that were expressed at a high level (Guo et al., 2013). Thus, we suggest that the DEGs in **Table 1** and **Figure 3** do not reflect physiologically relevant changes in gene expression associated with the Y610F directed mutation, as Y610F plants do not appear to be physiologically different from wild-type plants.

**FIGURE 4:**
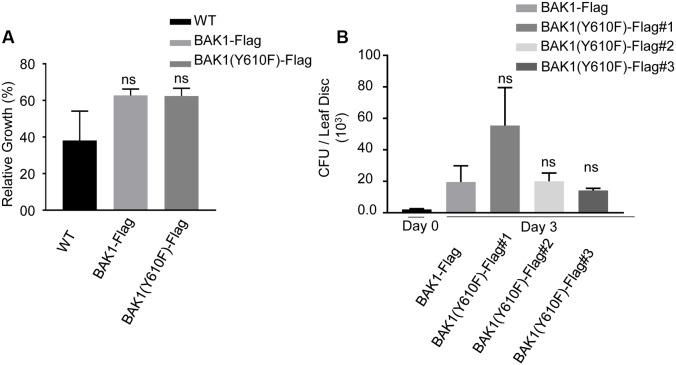
BAK1 (Y610F)-Flag plants respond similarly to wild-type and BAK1-Flag plants in FLS2-mediated inhibition of growth and bacterial growth following inoculation. **(A)** Relative growth inhibition in response to 10 nM flg22 in wild-type, BAK1-Flag and BAK1 (Y610F)-Flag. Each data point represents mean ± standard deviation (SD) of *n* = 7, Weltch *t*-test (*p* < 0.05) was performed, ns, not significant. **(B)** Bacterial growth measurement at 0 and 3 days post inoculation (dpi) of *hrpA^-^* mutant of *Pst* DC3000 in BAK1-Flag, BAK1 (Y610F)-Flag #1, #2, and #3 leaves. Bacterial suspension of 10^4^ cfu mL^-1^ was infiltrated into the abaxial leaf surface with a needleless syringe. Bacterial numbers were counted at 3 days post inoculation. Each bar represents mean ± standard deviation (SD) of *n* = 12 for BAK1-Flag, BAK1 (Y610F)-Flag #1, BAK1 (Y610F)-Flag #3 and *n* = 8 for BAK1 (Y610F)-Flag #2. Each sample consisted of four leaf disks of 5 mm diameter taken from inoculated leaves. Experiments were done three times with similar results.

BAK1 also functions as an important regulator of pathogen-associated molecular pattern (PAMP)-triggered immunity (PTI). In the presence of the bacterial PAMPs flg22 and elf18, BAK1 rapidly forms heteromers with FLS2 and EFR, respectively (Chinchilla et al., 2007; Heese et al., 2007; Schulze et al., 2010; Roux et al., 2011; Sun et al., 2013). This heteromerization evokes downstream PTI signaling which includes reactive oxygen species (ROS) production and mitogen-activated protein kinase (MAPK) cascades (Couto and Zipfel, 2016) and inhibits plant growth (Gomez-Gomez et al., 1999; Kunze et al., 2004). We compared wild-type plants and transgenic plants expressing BAK1-Flag or BAK1 (Y610F)-Flag in the flg22 seedling growth inhibition assay. As shown in **Figure 4A**, wild-type plants and transgenic plants expressing BAK1-Flag or BAK1 (Y610F)-Flag (in the double null mutant background) were equivalently and strongly inhibited by 10 nM flg22. These results suggest that phosphorylation of BAK1 at the Tyr-610 site is not required for BAK1 to initiate downstream PTI signaling. Further, we used those lines to re-evaluate earlier finding that the Y610F mutant allowed more growth of the non-pathogenic *hrpA^-^* mutant of *Pseudomonas syringae* pv. *tomato* (*Pst*) strain DC30000 in inoculated tissue. As shown in **Figure 4B**, although one BAK1 (Y610F)-Flag mutant line appeared in some assays more susceptible to *Pst* DC3000 *hrpA^-^*, we generally conclude that bacterial growth did not differ between the other BAK1 (Y610F)-Flag lines and the control BAK1-Flag. Thus, there is no indication that the Y610F plants have altered host defense responses at least in response to the bacterial strain tested. While no other biotic pathogens have been tested, we nonetheless conclude that the small differences in basal defense gene expression are not likely to be physiologically meaningful.

**FIGURE 5:**
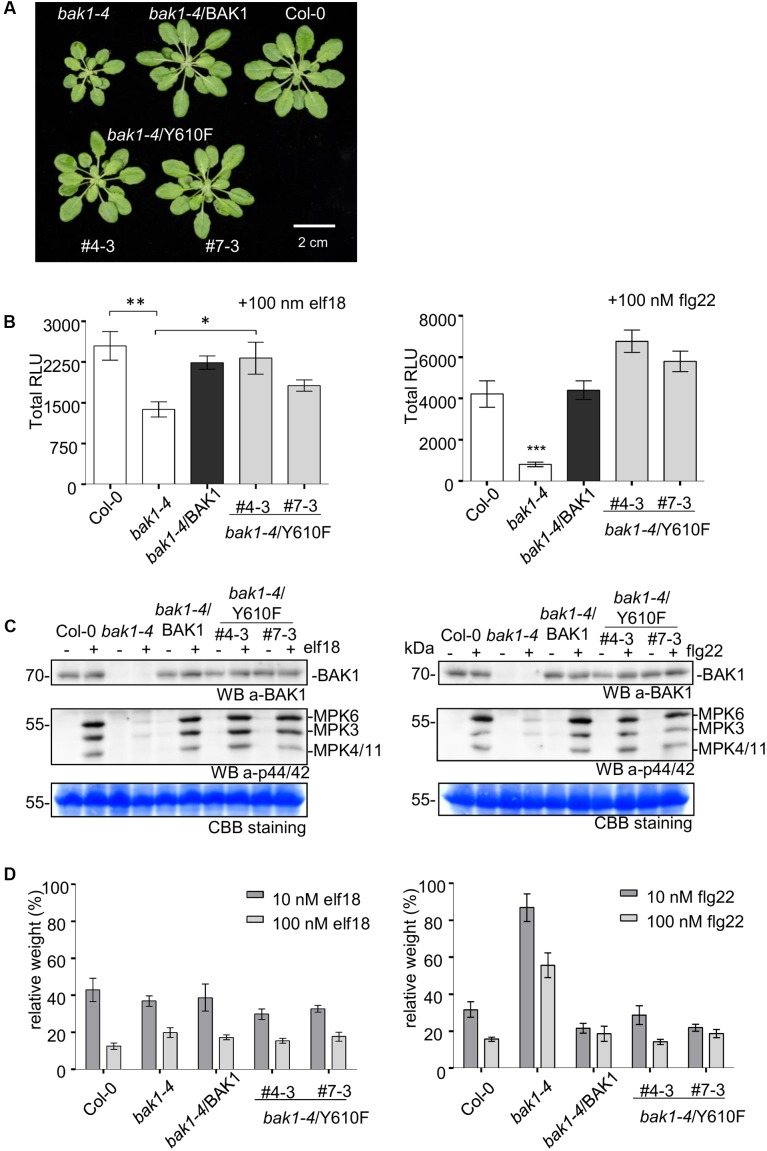
Stable expression of untagged BAK1 or BAK1 (Y610F) rescues the reduced leaf growth phenotype of the *bak1-4* mutant but does not affect EFR- and FLS2-dependent PTI signaling. **(A)** The *bak1-4*/BAK1 (Y610F) plants have a wild-type-like morphology under short-day conditions. Picture of representative individuals of 35-day-old Col-0, *bak1-4*, *bak1-4*/BAK1, *bak1-4*/BAK1 (Y610F)#4-3, and *bak1-4*/BAK1 (Y610F)#7-3 plants grown under short-day conditions. Scale bar represents 2 cm. **(B)** The BAK1 (Y610F) lines display wild-type levels of PAMP-induced ROS in stable Arabidopsis *bak1-4* mutant lines. Total ROS production in leaf disks of the indicated genotypes expressed as total relative light units (RLUs) after treatment with 100 nM elf18 (left panel) or 100 nM flg22 (right panel) for 60 min. Values are means ± SE (*n* = 12). Statistical analysis was performed using analysis of variance (ANOVA) and Bonferroni post-test (^∗∗∗^*p* < 0.001, ^∗∗^*p* < 0.01, ^∗^*p* < 0.05). **(C)** The BAK1 (Y610F) lines display wild-type levels elf18- (left panel) and flg22-induced (right panel) MAPK activation. Phosphorylation of MAPKs at 5 min after PAMP treatment, as shown by Western Blot using an anti-p44/42-ERK antibody. Individual MPKs are identified by molecular weight and indicated by arrows. The membranes were blotted with anti-BAK1 antibodies and subsequently stained with Coomassie colloidal blue for loading control. **(D)** BAK1 (Y610F) lines display wild-levels of elf18 (left panel) and flg22 (right panel)-induced seedling growth inhibition. Growth is represented as relative fresh weight to the untreated control. Results are means + SE (*n* = 8).

Previous studies revealed that several BAK1 fusion proteins with C-terminal tags (including Flag tag) strongly and specifically impair BAK1 function in PTI signaling (Ntoukakis et al., 2011). Thus, to better evaluate the function of BAK1 in PTI signaling, we generated transgenic lines of BAK1 and BAK1 (Y610F) in the *bak1-4* null mutant background, which shows reduced leaf growth (Kemmerling et al., 2007) and reduced ROS production in response to flg22 and elf18 (Chinchilla et al., 2007; Roux et al., 2011). We confirmed the previous finding that Y610F is not important for BR signaling, as the two independent transgenic lines *bak1-4*/BAK1 (Y610F) (#4-3 and 7-3) also rescued the growth defects of the *bak1-4* mutant under normal growth conditions (**Figure 5A**). We then examined whether BAK1 (Y610) is required for early and late PTI responses. We analyzed ROS production in response to 100 nM elf18 or flg22 and found that both BAK1 and BAK1 (Y610F) lines displayed similar levels of ROS production in response to elf18 (left panel) and flg22 (right panel; **Figure 5B**), while PAMP-mediated ROS production was strongly reduced in *bak1-4* in response to both elicitors. Treatments with PAMPs also rapidly activate MAP kinases including MPK3, MPK6, and MPK4/11 (Asai et al., 2002; Bethke et al., 2012). As shown in **Figure 5C**, treatment with 100 nM elf18 (left panel) or flg22 (right panel) for 5 min failed to strongly activate MPK3, MPK6, and MPK4/11 in the *bak1-4* mutant as expected but resulted in equivalent and robust MPK activation in Col-0 and transgenic plants expressing BAK1 or BAK1 (Y610F) in the *bak1-4* background. One late response induced by elf18 and flg22 is the growth inhibition of Arabidopsis seedlings. This can be easily measured as the reduction in growth of treated seedlings in comparison to untreated seedlings after 10 days of treatment with PAMPs (**Figure 5D**). Notably, BAK1 (Y610F) and BAK1 transgenic lines showed seedling growth inhibition similar to wild-type (Col-0) seedlings in response to elf18 and flg22. As expected, *bak1-4* had reduced growth inhibition in response to flg22 but not elf18 (Chinchilla et al., 2007; Roux et al., 2011) Based on these results we can conclude that transgenic plant expressing untagged BAK1 (Y610F) restores early and late responses to elicitors that are impaired in the null mutant *bak1-4*.

## Conclusion

Plants lack canonical tyrosine kinases, but many serine/threonine protein kinases are emerging as having dual specificity with phosphotyrosine function as an important mechanism. For example, BRI1 autophosphorylates on Tyr-831 (Oh et al., 2009), which appears to attenuate signaling activity (Oh et al., 2011), while trans-phosphorylation of BKI1 on Tyr-200 is essential to activation/initiation of BR signaling (Jaillais et al., 2011). Likewise, BAK1 trans-phosphorylation of BIK1 on multiple residues including tyrosine is essential to flg22 signaling and innate immunity (Lin et al., 2014), but we now show that autophosphorylation of Tyr-610 in BAK1 is not essential for its role in BR signaling, immune signaling or suppression of growth of at least one bacterial pathogen. It is entirely possible that Tyr-610, based on its location close to the carboxy-terminus of BAK1 and immediately adjacent to a PxxP motif (sequence: YPSGPR_COOH_) will have some functional role that is not yet apparent but the original report that this residue plays an essential role in BR or immune signaling is not correct.

## Author Contributions

VS selected and genotyped the BAK1-Flag and BAK1 (Y610F)-Flag plants in the double null *bak1 bkk1* background and performed the experiments; AP identified issues with previously published BAK1 (Y610F) lines, produced and characterized the untagged BAK1 transgenic plants in *bak1-4* and produced the results shown in **Figure 5**; SK performed preliminary experiments that laid the foundation for this work; BS initiated the generation of *bak1-4*/BAK1 plants and performed initial experiments in the CZ lab; VS, JL, and YZ performed the *Pst* DC3000 *hrpA^-^* inoculation experiment in **Figure 4B**; AM-C and SS assisted with qPCR and performed bioinformatics analysis of microarray results; M-HO performed the initial *Agrobacterium* transformation of D21 with Flag-tagged BAK1 and BAK1 (Y610F); CZ and SH conceived the study and oversaw research efforts. VS wrote the first draft of the manuscript which was edited by CZ and SH, prior to approval by all authors.

## Conflict of Interest Statement

The authors declare that the research was conducted in the absence of any commercial or financial relationships that could be construed as a potential conflict of interest.

## Footnotes

1 http://www.pnas.org/content/early/2016/06/17/1608778113.full
