# Supplementary material for: Tyrosine-610 in the Receptor Kinase BAK1 Does Not Play a Major Role in Brassinosteroid Signaling or Innate Immunity
Source: Front Plant Sci. 2017 Aug 2;8:1273. doi: 10.3389/fpls.2017.01273 (PMC5539094; doi:10.3389/fpls.2017.01273)
Supplement: Supplementary file 3 [file Table_3.DOCX]

Supplementary table 3

*List of primers used in this study*

| **Primer Name** | **Sequence** |
| --- | --- |
| BAK1 WT(GF2) | 5′-CCGCAGGACCACTTCTTTGATG-3’ |
| BAK1WT (R2) | 5′-CAAGTGGTGAGCTTAAGACATG-3’ |
| BAK1(F1) | 5′-TGGCCGATCTTGGCATTAAGTC-3’ |
| LB b1 | 5′- GCGTGGACCGCTTGCTGCAACT-3’ |
| BKK1 WT (LP) | 5′- TGGCTCAGAAGAAAACCACAG-3’ |
| BKK1 WT (RP) | 5′- CTGCTCCACTTCTGTTTCCAC-3’ |
| LCR67 RT F1 | 5′-GCTAAGTCTGCTACCATCGTTAC-3’ |
| LCR67 RT R1 | 5′-CATGGGAAGTAGCAGATACATTTG-3’ |
| PCC1 RT F1 | 5′-CCGCGCAAAATTACTTTTC-3’ |
| PCC1 RT R1 | 5′-CTCTGATGTACAGAGGCTGG-3’ |
| BT5 RT F | 5′-ATTTCAAGCGTGTAATGGTT-3’ |
| BT5 RT R | 5′-TCTCATCTTTCCTGCTCTGT-3’ |
| At5g39180 RT F | 5′-CAAGTGAATATCGGGAAGAC-3’ |
| At5g39180 R | 5′-GTTTTTAAACTTGGCCTCAA-3’ |
| ACTIN2 F | 5′-ATGCCATCCTCCGTCTTGAC-3’ |
| ACTIN2 R | 5′-CGCTCTGCTGTTGTGGTGAA-3’ |
| BAK1 epiGreen_F | 5’-GTTACTCCATGTACATGGTTTCATGTTACTTGCAATAGC-3’ |
| BAK1 epiGreen_R | 5’-TGACTCTAGAGGATCCTTATCTTGGACCCGAGGGGTATTC-3’ |
| BAK1(Y610F) epiGreen_R | 5’-TGACTCTAGAGGATCCTTATCTTGGACCCGAGGGGAATTC-3’ |
